# Effects of a 24-week exercise program on anthropometric, body composition, metabolic status, cardiovascular response, and neuromuscular capacity, in individuals with intellectual and developmental disabilities

**DOI:** 10.3389/fphys.2023.1205463

**Published:** 2023-05-23

**Authors:** Miguel Jacinto, Rui Matos, Diogo Monteiro, Raul Antunes, André Caseiro, Beatriz Gomes, Maria João Campos, José Pedro Ferreira

**Affiliations:** ^1^ Faculty of Sport Sciences and Physical Education, University of Coimbra, Coimbra, Portugal; ^2^ ESECS—Polytechnic of Leiria, Leiria, Portugal; ^3^ Life Quality Research Centre (CIEQV), Leiria, Portugal; ^4^ Research Center in Sport Sciences, Health Sciences and Human Development (CIDESD), Vila Real, Portugal; ^5^ Center for Innovative Care and Health Technology (ciTechCare), Polytechnic of Leiria, Leiria, Portugal; ^6^ Research Center for Sport and Physical Activity (CIDAF), Coimbra, Portugal

**Keywords:** cardiorespiratory training, health variables, indoor training, outdoor training, resistance training, strength capacity

## Abstract

**Introduction:** The prevalence of overweight and obesity has increased in the last decades, including in people with Intellectual and Developmental Disabilities (IDD). This is even more concerning when it is globally accepted that a low physical condition contributes to the deterioration of functionality and increases the risk of developing chronic diseases during life, with effective implications for health and well-being. The aim of the present study is to investigate the effects of two physical exercise intervention programs on institutionalized individuals with IDD.

**Methods:** Twenty-one adults with IDD (43.04 ± 11.18 years) were split by convenience into three groups: i) an indoor training group (IG; N = 7; 24-week machine-based gym intervention), ii) an outdoor training group (OG; N = 7; 24-week outdoor intervention with low-content materials), and iii) a control group (CG; N = 7). Assessed outcomes included indicators of health and neuromuscular capacity. The *ShapiroWilk* (*n* < 50) and *Levene* tests were used to verify data normality and homoscedasticity. A *Kruskal-Walli* test was performed to understand if there were differences between the groups. For comparison purposes and to assess hypothetical differences between groups, the *Wilcoxon* signed-rank test and the *Friedman* test were used. The respective effect size was calculated, and the significance level was defined at 0.05.

**Results/Discussion:** There was a difference in fat mass in OG (initial ≠ intermediate; *Bonferroni* corrected: *t* = 2.405; *p* = 0.048; *W* = 0.08 and initial ≠ final moments; *Bonferroni* corrected: *t* = 2.405; *p* = 0.048; *W* = 0.08). Indoor intervention programs seem to be more effective than outdoor intervention programs for reducing heart rate rest (*t* = −2.912; *p* = 0.011; *W* = −0.104) when compared with CG.

**Conclusion:** A low-cost outdoor intervention in contact with nature appears to be more effective for fat mass reduction. The results for heart rate variability are not clear and robust. Finally, an indoor intervention using weight-training machines appears to be a good method to promote neuromuscular capacity.

## 1 Introduction

Intellectual and Developmental Disability (IDD) is defined as a deficit in intellectual and adaptive functioning in the conceptual, social, and practical domains, which can be identified with mild, moderate, severe, and profound degrees and develops before 22 years old (Schalock et al., 2021). Several studies show that this population has a higher prevalence of hypertension, obesity, hypercholesterolemia, diabetes type II, and metabolic syndrome compared to the disabled population (Krause et al., 2016). At the same time, studies show that hypertension, dyslipidemia, obesity, diabetes, and impaired glucose metabolism are associated with lower heart rate variability (HRV) (Coopmans et al., 2020; Maciorowska et al., 2020). In individuals with IDD, the functioning of the autonomic nervous system seems to be impaired (Zwack et al., 2021), as well as low HRV values (Chang et al., 2012), which can be related to high blood glucose levels that lead to damage to peripheral nerve fibers, increasing sympathetic activity and decreasing parasympathetic activity (Chang et al., 2012; Font-Farré et al., 2021). All these comorbidities are considered cardiovascular and metabolic risk factors, which, in turn, are associated with an increased risk of premature death (O’Leary et al., 2018).

To decrease the risk of cardiovascular and metabolic diseases, medications are often prescribed (O’Dwyer et al., 2016), increasing healthcare spending instead of adopting healthy and active lifestyles. Besides being inactive, people with IDD are a mostly sedentary population (Dairo et al., 2016), not complying with recommendations for health maintenance or gains (World Health Organization, 2020). The latest recommendations are for adults to participate in at least 150–300 min of moderate-intensity physical activity per week or 75–150 min of vigorous-intensity physical activity per week. Individuals with IDD should also participate in exercise sessions that focus on flexibility, aerobic capacity, and endurance that involve all major muscle groups on at least 2 days per week (American College of Sports Medicine, 2021) for the maintenance/development of functional independence.

As a result of an inactive and sedentary lifestyle, it is thus evident that individuals with IDD have reduced values in all physical fitness, including strength (Borji et al., 2014; Wouters et al., 2020). Similarly, this loss of muscle strength is strongly associated with a decline in physical and functional capacity (Carmeli et al., 2012), health, and life expectancy (Zghal et al., 2019). Muscle strength has been associated with cardiovascular risk, showing that higher levels of strength are associated with decreased waist circumference (WC) and triglycerides in children, adolescents, and adults (De Lima et al., 2021). In fact, the higher levels of upper and lower muscle strength observed in adults are associated with a lower risk of mortality (Garcia-Hermoso et al., 2020). On the other hand, regular exercise has been shown to be an effective method for improving functionality, cardiorespiratory capacity, mobility, performance in activities of daily living and mental health, and decreasing the risk of cardiovascular and metabolic diseases (Jacinto et al., 2021a; Jacinto et al., 2021b; Obrusnikova et al., 2021).

One of the reasons found in the literature for increased physical inactivity and sedentary lifestyles in individuals with IDD is the existence of barriers to promoting regular practice, namely, the lack of adapted exercise programs for individuals with IDD, limited financial resources, and a lack of places to exercise (Jacinto et al., 2021c). On the other hand, there is a scarcity of research, little clarity in intervention protocols, or diverse methodologies that approach the applicability of non-pharmacological, psychologica,l and psychosocial interventions, such as exercise programs for the improvement of the health-related variables that may be related to cardiovascular and metabolic diseases (Sheehan et al., 2014).

The prescription and implementation of exercise programs that decrease the mentioned barriers to practice become fundamental to achieving clinically demonstrated and significant health benefits in the variables (Bartlo and Klein, 2011). On the other hand, in addition to promoting the practice and adherence to exercise programs, it may delay the functional and physical decline of individuals with IDD, which begins around the age of 40–50 years old, which, combined with improved medical and healthcare, may promote quality of life (Schalock et al., 2002).

To our knowledge, this will be the first study to prescribe and implement exercise programs in different contexts, assessing their impact on several aspects of physical health and fitness. It is an attempt to present an effective strategy/tool for decreasing barriers to physical exercise and promoting these variables. Therefore, the present non-randomized experimental study aimed to assess the effects of two 24-week exercise programs (indoor and outdoor/low cost) on anthropometric, body composition, metabolic status, cardiovascular response, and neuromuscular capacity in institutionalized individuals with IDD. For the present study, the following hypotheses were defined: i) the indoor group (IG) significantly improved all variables assessed after a 12-week exercise program; ii) the IG significantly improved all variables assessed after a 24-week exercise program; iii) Compared to the outdoor group (OG), the IG significantly improved all variables assessed after a 12-week exercise program; iv) Compared to the OG, the IG significantly improved all variables assessed after a 24-week exercise program; v) Compared to the control group (CG), IG significantly improved all variables assessed after a 12-week exercise program; vi) Compared to CG, IG significantly improved all variables assessed after a 24-week exercise program; vii) OG significantly improved all variables assessed after a 12-week exercise program; viii) OG significantly improved all variables assessed after a 24-week exercise program; ix) Compared to IG, OG significantly improved all variables assessed after a 12-week exercise program; x) Compared to IG, OG significantly improved all variables assessed after a 24-week exercise program; xi) Compared to CG, OG significantly improved all variables assessed after a 12-week exercise program; xii) Compared to CG, OG significantly improved all variables assessed after a 24-week exercise program.

## 2 Methods

### 2.1 Research design

This study follows a non-randomized experimental design, according to the Helsinki Declaration (World Medical Association, 2013) and (Harriss et al., 2019) and the Standards for Ethics in Sport and Exercise Science Research was approved by the Ethics Committee of the Faculty of Sport Sciences and Physical Education - University of Coimbra, with the approval code CE/FCDEF-UC/00872021. All subjects and their families were informed of the purpose and methods of the experimental methodology and signed an informed consent form. The total methodology was presented in a preliminarily published study protocol (Ferreira et al., 2022).

### 2.2 Participants

The experimental study recruited adults volunteer, institutionalized in a support Institution, placed in Leiria. Inclusion criteria were defined as 1) adults with IDD; 2) no medical contraindications for adherence to a physical activity program; 3) 18 years old or older; 4) participants diagnosed with mild, moderate, or severe IDD diagnosis (Down Syndrome inclusive); 5) successful in performing movements such as pulling/pushing; and 6) the ability to carry out the intended assessments. Exclusion criteria were defined as 1) subjects who can not commit for 24 weeks; 2) subjects with other associated pathologies; 3) incapacity to walk unassisted; 4) profound IDD; 6) inability to communicate; and 7) failure to provide a duly signed informed consent form.

Due to the characteristics of this population and logistical constraints inherent in the development of intervention studies, the sample consisted of the first 21 participants who agreed to participate in the program, aged between 18 and 65 years (10 females, 11 males,*M* age = 43.04; *SD* = 11.18 years old). A power analysis (calculated using G*Power, version 3.1.9.7 (Faul et al., 2007) showed that a sample of at least 15 was required to detect a medium effect size (*ES*) of 0.5 (*α* = .05, 1 − *β* = 0.95) using a repeated-measures analysis of variance (*ANOVA*), in agreement with some previous studies (Cicone et al., 2018; Fujita et al., 2021). An effect size of 0.5 was chosen given that this value was confirmed from studies that explored the effects of exercise on the variables of interest in our study (Bartlo and Klein, 2011; John et al., 2020; Obrusnikova et al., 2021).

### 2.3 Materials/instruments

#### 2.3.1 Anthropometry

Portable stadiometer seca (model 870) scale was used to measure body weight and height. Body mass index (BMI) was calculated using the weight (kg)/height (m^2^) formula and the WC was assessed with a flexible measuring tape directly on the skin from the middle of the iliac crest and the 10th rib. All these methods were viable, reliable, and accurate for IDD participants (Temple et al., 2010; Casey, 2013; Wouters et al., 2017; Oppewal and Hilgenkamp, 2018).

#### 2.3.2 Body composition

A bioelectrical impedance device (InBody770) was used to measure body composition, being a viable, reliable, and non-invasive method (Havinga-Top et al., 2015; Wouters et al., 2017; Patusco et al., 2018). The variables that were assessed were WC, body weight, BMI, fat mass, and muscle mass.

#### 2.3.3 Metabolic status

To assess metabolic status, blood samples were collected (glycemia, cholesterol, and triglycerides) by accredited specialists using the venous point technique (World Health Organization, 2010). The results were analyzed in a certified laboratory and sent by email to the main study investigator.

#### 2.3.4 Cardiovascular response

The digital sphygmomanometer Omron Digital Blood Pressure Monitor HEM-907 (Omron Healthcare Europe BV, Matsusaka, Japan) was used to measure hemodynamic parameters such as resting blood pressure (systolic (SBP) and diastolic (DBP)) and resting heart rate (HRrest). Before data collection, participants remain completely at rest for 5 minutes, legs uncrossed, back and arms supported without talking and/or moving (Muntner et al., 2019). Two measurements were taken with an interval of 1–2 min and the average of these readings was recorded. If the values deviate ≥ 5 mmHg, a third measurement was taken (Muntner et al., 2019). Measurements were taken in the morning after breakfast, and participants were instructed to avoid caffeine, exercise, and smoking at least 30 min before the measurement (Muntner et al., 2019).

Heart rate variability was also measured according to Proietti (Proietti et al., 2017) and (Task Force of the European Society of Cardiology and the North American Society of Pacing and Electrophysiology, 1996) guidelines, with the Polar ProTrainer (Kempele, Finland). Participants had the sensor on their chest under the pectoralis major muscle. Participants were instructed to sit comfortably in a chair, keep their eyes open, breathe quietly, and avoid movement during data collection. The test took 10 min in a quiet, silent, and low-light environment. After the test was completed, the data was downloaded via *the Polar Flow Web Service* as “.txt” files and exported for analysis using the *Kubios HRV* software (Kubios HRV, Biomedical Signal Analysis Group, Department of Applied Physics, University of Kuopio, Finland) (Tarvainen et al., 2014). The RR intervals corresponding to the first 2 min were discarded (stabilization period) and data from the next 5 min was used to calculate HRV. The following items were calculated in the time domain: i) mean RR (mean of the RR intervals in ms); ii) SDNN (standard deviation of RR intervals in ms); iii) RMSSD (root mean square of consecutive RR intervals in ms); and iv) pNN50 (percentage of successive RR intervals that differ by more than 50 m). The following items were calculated, in the frequency domain: i) LF (absolute power of the low-frequency band, 0.04–0.15 Hz, in ms2); ii) HF (absolute power of the high-frequency band, 0.15–0.4 Hz, in ms2; iii) ratio of LF-to-HF power (LF/HF).

#### 2.3.5 Neuromuscular capacity

Neuromuscular capacity (peak torque) was measured using an isokinetic dynamometer (BIODEX Multijoint System 3 Pro), which is reliable for the target population (Pitetti, 1990), by testing maximal concentric knee flexion and knee extension at an angular velocity of 60 and 120 °/s. Equipment calibration was performed before the assessment session according to the manufacturer’s instructions (Biodex Medical Systems, Inc., 2000).

A manual dynamometer was used to perform a handgrip test to assess upper limb strength measurement. The test reliability and validity were confirmed by Cabeza-Ruiz (Cabeza-Ruiz et al., 2019) and Oppewal and Hilgenkamp (Oppewal and Hilgenkamp, 2020) and the protocol used was recommended in the *Brockport Fitness Test Manual* (Winnick and Short, 2014).

The “3 kg medicine ball throw test” was used (Harris et al., 2011) to assess the muscular power of the upper limbs since it is a valid and reliable test for people with IDD (Lencse-Mucha et al., 2015; Wouters et al., 2017; Aertssen et al., 2018; Tatar, 2018). The participant was seated in a chair with the ball held close to their chest. At the starting signal, the participant threw the ball, using a chest pass, as far as possible.

### 2.4 Procedure

All the participants were assessed at the Faculty of Sport Sciences and Physical Education–University of Coimbra, using specific and valid test protocols to assess individuals with IDD. The space was large and isolated, the temperature was controlled, and each stage of the assessment was organized as comfortably and privately as possible for all participants. The researchers provided information about the procedures and objectives for each test and were available to answer questions at any time.

During the exercise program, three assessments were carried out (initial, week −1; intermediate, week 12; final, week 24). To minimize procedural differences, tests were carried out using the same research team at all times. Additionally, all assessments were carried out in a controlled environment, in the morning, after fasting, due to the specific characteristics of the participants and their medication needs.

Participants were allocated to one of the three groups: i) IG with sessions carried out in a gym, using weight machines; ii) OG with outdoor sessions using low-cost materials; and iii) CG with participants continuing to do their normal activities. Interventions with physical exercise were based on a 24-week combined exercise program offered twice a week for approximately 45 min each session. The exercise program was conducted following the guidelines recommended by the American College of Sports Medicine (Bayles, 2023). The attendance of the participants was calculated, revealing that the IG and OG showed attendance average values of 78% and 76%, respectively. The CG participants were encouraged to continue their usual lifestyle throughout the period of 24 weeks.

### 2.5 Intervention

All the exercise sessions were conducted and supervised by two instructors who had graduated in sports science with expertise regarding people with disabilities, specifically those with IDD. Both instructors encouraged participants to complete their exercise sessions and provided instruction and demonstration. Feedback was adjusted to assure correction and safety during the execution of the exercises. In both interventions, all sessions were structured following the phases: warm-up (5–7 min), the main part of the session (aerobic plus resistance exercise, 33–35 min), and static stretches or return to calm (5 min), with a progressive load method. Participants from both groups were encouraged to conduct their daily activities as usual besides the intervention and to maintain the same nutritional pattern/diet. Intensity of the interventions were controlled by heart rate reserve, Borg RPE scale (Borg, 1982) or Borg CR-10 Scale (Borg, 1998).

#### 2.5.1 Indoor training program

The indoor exercise program was carried out in a gym using weight machines for resistance training (Table 1).

**TABLE 1:** Indoor training program.

Exercise	Duration	Speed	Intensity	Rep’s	Series	Volume (min)
“Caterpillar game” or shuttle run	5–7 min					40
Treadmill	10 min	Walk	40%–80% of Heart Rate Reserve; 12 to 17 according to the Borg RPE Scale (Borg, 1982); between 5 and 8 according to the Borg CR-10 Scale (Borg, 1998)			
Leg press	23–25 min	Max. concentric. Eccentric 3 s	40%–80% of 3 RM	10–15	2–3	
Chest press						
Leg extension						
Lat Pull Down						
Leg curl						
Shoulder press						

Flexibility: static method (stretching)				
Exercise	Duration (second)	Series	Recovery	Volume
4 static stretches	30 s	1	Does not exist	2 min

Note: the rest interval between exercises or sets is 30 s

Note: stretching performed unilaterally, should last 15 s per limb.

#### 2.5.2 Outdoor training program

The outdoor exercise program was carried out in a natural environment near the institution (Table 2). Natural environments are defined, for the purpose of this experimental study, as “any outdoor spaces with elements of nature, from pure or semi-natural areas to urban green or blue spaces, including green infrastructure” (Brito et al., 2022).

**TABLE 2:** Outdoor training program.

Exercise	Duration	Speed	Intensity	Rep’s	Sets	Volume (min)
Caterpillar game or shuttle	5–7 min					40
Walk	10 min	March	40%–80% of Heart Rate Reserve; 12 to 17 according to the Borg RPE Scale (Borg, 1982); between 5 and 8 according to the Borg CR-10 Scale (Borg, 1998)			
Stand up/sit down from chair + elastic bands	23–25 min	Max. Concentric eccentric 3s	6–9 OMNI-RES scale	15	3	
Low row + elastic bands						
Unilateral knee extension + shin guards						
Chest press + elastics						
Single leg curl + shin guards						
High row + elastic						

Flexibility: static method (stretching)				
Exercise	Duration (second)	Series	Recovery	Volume
4 static stretches	30 s	1	Does not exist	2 min

Note: the rest interval between exercises or sets is 30 s

Note: stretching performed unilaterally, should last 15 s per limb.

### 2.6 Statistical analysis

Descriptive statistics including mean and standard deviation were calculated for the studied variables. Normality and homoscedasticity data were verified by *Shapiro–Wilk* (*n* < 50) and *Levene* tests. Thus, a *Kruskal–Wallis* test was performed to understand if there were differences between groups. The *Wilcoxon* signed-rank test and *Friedman* test were used for the comparison and identification of possible differences in each group. Both these tests are non-parametric *ANOVAs* and were adjusted for small sample testing. The multiple comparison test used the *Bonferroni* correction (i.e., alpha level/number of tests) to avoid error Type I (Ho, 2014). The effect size *η* ^*2*^ (suitable for the *Wilcoxon* test, allowing comparison of two paired groups) was calculated and the assumed reference values were: “small” effect ≥ 0.01, “medium” effect ≥ 0.3, and “large” effect ≥ 0.5 (Cohen, 1988; Fritz et al., 2012). Therefore, *Kendall’s W* effect size (suitable for the *Friedman* test, allowing comparison of two paired groups) was calculated and the assumed reference values were as follows: “small” effect ≥ 0.01, “medium” effect ≥ 0.3, and “large” effect ≥ 0.5 (Cohen, 1988; Fritz et al., 2012). The significance level for rejecting the null hypothesis was set at 5% and the analysis was performed in IBM SPSS.

## 3 Results

Table 3 presents descriptive statistics, namely, group means and standard deviation values regarding anthropometry and body composition values (initial, intermediate, and final assessments).

**TABLE 3:** Global sample description for anthropometry and body composition values.

	IG			OG			CG		
	Mean ± standard deviation								
	Initial	Intermediate	Final	Initial	Intermediate	Final	Initial	Intermediate	Final
WC (cm)	93.18 ± 12.95	93.04 ± 12.12	92.45 ± 11.92	90.37 ± 16.72	89.52 ± 16.49	89.42 ± 16.49	93.55 ± 15.81	93.28 ± 16.56	95.57 ± 18.15
Body weight (kg)	73.87 ± 11.75	73.41 ± 12.08	73.35 ± 11.33	75.21 ± 19.86	74.44 ± 19.3	73.55 ± 18.81	73.27 ± 18.19	73.4 ± 18.86	73.65 ± 18.71
BMI (kg/m^2^)	28.82 ± 5.53	28.81 ± 5.67	29.5 ± 5.57	30.34 ± 8.7	30 ± 8.42	29.65 ± 8.24	28.24 ± 6.87	28.27 ± 7.12	28.41 ± 7.02
Fat mass (kg)	27.08 ± 11.31	25.87 ± 11.83	28.5 ± 11.83	30.11 ± 17.87	29.08 ± 17.52	28.62 ± 17.06	27.22 ± 14.09	26.78 ± 14.7	27.69 ± 14.55
Muscle mass (kg)	25.74 ± 4.16	26.28 ± 3.88	26.02 ± 4.07	24.78 ± 5.08	25.04 ± 4.88	24.85 ± 4.8	25.2 ± 3.97	25.48 ± 4.06	24.93 ± 4.35

BMI, body mass index; IG, indoor group; OG, outdoor group; CG, control group; WC, waist circumference.

Table 4 presents the descriptive statistics for the cardiovascular response, taking into consideration the three assessments for the three groups.

**TABLE 4:** Global sample description for cardiovascular response.

	IG			OG			CG		
	Mean ± standard deviation								
	Initial	Intermediate	Final	Initial	Intermediate	Final	Initial	Intermediate	Final
SBP (mm Hg)	116.71 ± 10.85	112.71 ± 15.17	102.67 ± 16.44	127.57 ± 12.52	119.57 ± 13.16	116.43 ± 12.89	129 ± 28.15	128.43 ± 27.79	128.43 ± 29.38
DBP (mm Hg)	73.29 ± 7.85	76.43 ± 7.32	76 ± 10.11	81.71 ± 15.76	78.57 ± 13.17	79.14 ± 9.24	84.86 ± 8.68	85.29 ± 9.81	87 ± 10.23
HRrest (mm Hg)	66.71 ± 7.97	70 ± 10.34	66.71 ± 8.86	76.14 ± 18.06	76 ± 16.98	74.14 ± 19.29	80 ± 12.91	82.57 ± 9.18	85 ± 8.83
Mean RR (ms)	878.80 ± 147.99	616.29 ± 156.34	837.84 ± 198.43	832.30 ± 181.12	843.21 ± 160.34	779.56 ± 227.09	722.30 ± 132.17	608.320 ± 116.10	512.91 ± 353.87
SDNN (ms)	39.27 ± 14.36	43.55 ± 30.53	40.28 ± 25.98	40.28 ± 25.98	45.65 ± 31.95	32.76 ± 21.67	27.61 ± 17.17	29.99 ± 17.68	28.2 ± 14.69
Mean HR (ms)	69.76 ± 10.53	101.94 ± 21.24	74.48 ± 14.7	75.7 ± 19.96	73.82 ± 16.63	82.42 ± 22.44	85.08 ± 12.95	101.29 ± 16.53	92.12 ± 16.4
RMSSD (ms)	27.76 ± 10.77	15.75 ± 9.2	23.68 ± 14.59	29.21 ± 15.29	33.23 ± 27.98	19.63 ± 15.36	20.17 ± 20.81	15.84 ± 16.8	17.12 ± 16.07
pNNxx (%)	7.73 ± 8.55	1.01 ± 1.35	8.07 ± 14.25	10.51 ± 10.66	11.19 ± 15.92	5.36 ± 10.79	2.2 ± 5.29	2.83 ± 6.93	2.84 ± 6.95
LF log	5.6 ± 1.17	4.76 ± 1.63	5.32 ± 1.16	5.96 ± 1.19	5.87 ± 1.78	5.68 ± 1.57	5.12 ± 1.27	4.88 ± 1.36	4.55 ± 1.96
HF log	5.12 ± 1.13	3 ± 1.53	4.79 ± 1.40	5.47 ± 1.59	4.79 ± 2.44	4.24 ± 1.63	4.57 ± 1.58	2.57 ± 1.89	3.73 ± 1.97
LF n.u	57.96 ± 21.67	80.75 ± 21.63	63.26 ± 10.9	57.94 ± 27.75	62.53 ± 23.8	77.74 ± 12.64	60.41 ± 23.59	76.09 ± 16.64	57.82 ± 28.09
HF n.u	41.87 ± 21.81	19.16 ± 21.51	36.7 ± 10.88	41.87 ± 21.81	37.45 ± 23.79	22.21 ± 12.62	39.51 ± 23.52	23.78 ± 16.52	31.99 ± 16.14
LF/HF ratio	2.05 ± 1.61	8.77 ± 6.48	1.89 ± .69	4.18 ± 6.2	3.27 ± 3.67	6.14 ± 5.69	3 ± 3.31	5 ± 3.72	2.57 ± 2.7

IG, indoor group; OG, outdoor group; CG, control group; SBP, systolic blood pressure; DBP, diastolic blood pressure; HR_rest_, heart rate rest; mean RR, mean of the RR intervals; SDNN, standard deviation of RR intervals; RMSSD, root mean square of consecutive RR intervals; pNN50, percentage of successive RR intervals that differ by more than 50 m; LF, absolute power of the low-frequency band, 0.04–0.15 Hz, in ms2; HF, absolute power of the high-frequency band, 0.15–0.4 Hz, in ms2; LF-to-HF power (LF/HF).

Through the collection of blood tests, the variables blood glucose, cholesterol, and triglycerides were analyzed in two different assessments (initial and final). The descriptive statistics values for metabolic status are presented in Table 5.

**TABLE 5:** Global sample description for metabolic status.

	IG		OG		CG	
	Mean ± standard deviation					
	Initial	Final	Initial	Final	Initial	Final
Glycemia (mg/dL)	84.28 ± 17.06	85.5 ± 13.85	102.71 ± 24	100.85 ± 24.63	101.71 ± 30.4	100.57 ± 31.74
Cholesterol (mg/dL)	174 ± 42.27	170.07 ± 45.38	207.85 ± 35.96	201.85 ± 31.29	188.71 ± 51.11	191.1 ± 69.75
Triglycerides (mg/dL)	120.42 ± 42.16	127.83 ± 53.2	124.14 ± 51.83	123.71 ± 50.82	167 ± 99.96	166.71 ± 109.01

IG, indoor group; OG, outdoor group; CG, control group.

In Table 6, it can be observed the mean and standard deviation values of the neuromuscular capacity variables, namely, the peak torque of both lower limbs at 60°/s and 120°/s.

**TABLE 6:** Global sample description of the neuromuscular capacity variables.

	IG			OG			CG		
	Mean ± standard deviation								
	Initial	Intermediate	Final	Initial	Intermediate	Final	Initial	Intermediate	Final
PT cc KE 60°/s right (N.M)	67.35 ± 30.88	78.7 ± 40.24	76.7 ± 16.65	64.24 ± 59.59	82.65 ± 64.77	73.78 ± 65.44	45.41 ± 23.01	57.57 ± 22.71	49.98 ± 33.37
PT cc KE 60°/s left (N.M)	81 ± 25.47	80.84 ± 28.76	91.4 ± 27.36	69.7 ± 68.7	67.1 ± 67.22	76.92 ± 59.33	53.85 ± 26.49	59 ± 31.04	46.5 ± 35.1
PT cc KF 60°/s right (N.M)	37.5 ± 22.47	37.95 ± 26.17	37.85 ± 13.21	33.34 ± 30.31	46 ± 38.69	37.14 ± 38.2	14.32 ± 8.66	21.31 ± 7.88	17.27 ± 11.88
PT cc KF 60°/s left (N.M)	43.62 ± 22.31	51.48 ± 21.58	49.5 ± 8.82	31.6 ± 28.82	41.64 ± 34.5	37.64 ± 33.86	18.02 ± 11.91	23.5 ± 12.95	15.41 ± 9.5
PT cc KE 120°/s right (N.M)	55.48 ± 23.76	58.82 ± 32.45	61.57 ± 17.14	50.42 ± 56.22	60.31 ± 51.35	53.18 ± 53.99	36.22 ± 11.39	36.98 ± 21.11	40.95 ± 23.5
PT cc KE 120°/s left (N.M)	60.84 ± 26.77	59.14 ± 26.89	68.7 ± 23.35	51.31 ± 58.71	55.82 ± 55.39	54.92 ± 51.13	41.5 ± 15.2	38 ± 22.32	48.15 ± 32.25
PT cc KF 120°/s right (N.M)	32.65 ± 13.93	34.02 ± 20.06	30.52 ± 9.71	28.11 ± 31.29	34.28 ± 32.14	31.27 ± 37.70	10.15 ± 7	14.38 ± 7.92	18.48 ± 7.70
PT cc KF 120°/s left (N.M)	30.85 ± 21.27	39.42 ± 24.95	38.67 ± 4.78	27.32 ± 29.77	27.87 ± 28.99	29.14 ± 31.64	12.26 ± 6.45	18.62 ± 16.26	17.7 ± 6.22
3 kg medicine ball throw (m)	2.36 ± 0.68	2.6 ± 0.55	2.6 ± 0.36	2.3 ± 1.21	2.6 ± 1.33	3.2 ± 1.83	2.14 ± 0.68	2.1 ± 0.62	1.9 ± 0.73
manual dynamometer (kg)	24.02 ± 4.08	25.25 ± 5.76	26.05 ± 5.63	20.27 ± 10.25	20.68 ± 11.59	24.28 ± 11.37	15.9 ± 5.49	15.52 ± 5.6	15.01 ± 5.84

IG, indoor group; OG, outdoor group; CG, control group; PT, peak torque; cc, concentric; KE, knee extension; KF, knee flexion.

In Table 7, it can be observed the differences between groups and assessments for anthropometry and body composition. No significant statistical differences were found for the anthropometry and body composition variables between groups at the initial assessment as well as at both the intermediate and final assessments.

**TABLE 7:** Difference between groups and assessments for anthropometry and body composition values.

	IG			OG			CG			Intermediate				Final				
	Median (interquartile range)									*t* ^a^	*df* ^a^	*p* ^a^	Pairwise comparisons (groups)^b,c^	*t* ^a^	*df* ^a^	*p* ^a^	Pairwise comparisons (groups)^b,c^	Pairwise comparisons (moments)^b,c^
	Initial	Intermediate	Final	Initial	Intermediate	Final	Initial	Intermediate	Final									
WC (cm)	89.5 (24.5)	90.4 (22.7)	90.5 (22)	96 (36)	95 (35.4)	97 (34)	91.6 (30.1)	90.4 (32.2)	92 (36)	.139	2	*p* > 0.05	d	.248	2	*p* > 0.05	d	d
Weight (kg)	76.9 (18.2)	77.3 (22.4)	78.4 (22)	75.5 (35.4)	73 (33.7)	71.9 (29.6)	72 (34.1)	72 (36.5)	69.3 (40.9)	.002	2	*p* > 0.05	d	.030	2	*p* > 0.05	d	d
BMI (kg/m^2^)	29.3 (7.3)	30 (16.5)	29.55 (6.8)	29.2 (19.1)	28.8 (18.5)	28.1 (17.8)	26.5 (13.2)	26.5 (13.4)	25.5 (14.3)	.141	2	*p* > 0.05	d	.119	2	*p* > 0.05	d	d
Fat mass (kg)	29 (23.6)	29.5 (24.8)	30.8 (24.6)	27.3 (29.9)	26.7 (30.2)	25.2 (28.1)	25 (22.6)	23.3 (22.7)	29.35 (26)	.247	2	*p* > 0.05	d	.035	2	*p* > 0.05	d	initial ≠ intermediate (OG); initial ≠ final (OG)
Muscle mass (kg)	24.4 (6.4)	26 (5.2)	25.2 (5.35)	23.5 (10.9)	24.6 (8.5)	23.7 (8.6)	24.9 (7.7)	25.2 (8.1)	24.3 (9.5)	.23	2	*p* > 0.05	d	.459	2	*p* > 0.05	d	d

IG, indoor group; OG, outdoor group; CG, control group; *t*, test statistic; *df*, degrees of freedom; a, *Kruskal–Wallis*; b, *Friedmann*; c, *Bonferroni correction*; d, No differences detected.

A significant difference was found between assessments for fat mass on OG (OG (initial ≠ intermediate; Bonferroni corrected: *t* = 2.405; *p* = 0.048; *W* = 0.08 and initial ≠ final; Bonferroni corrected: *t* = 2.405; *p* = 0.048; *W* = 0.08).

In Table 8, it can be observed the differences between groups and assessments for cardiovascular response. At the initial and intermediate assessments, there were no significant differences between groups for the blood pressure and heart rate variability variables (*p* ≥ 0.05). Significant statistical differences were found between groups for the HRrest variable (*p* = 0.014) at the final assessment. After Bonferroni correction, these differences were observed between IG and CG (*t* = −2.912; *p* = 0.011; *W* = −0.104). There were also significant differences between assessments when considering the CG (initial ≠ final moment; *Bonferroni* corrected: *t* = −2.405; *p* = 0.048; *W* = 0.17).

**TABLE 8:** Difference between groups and assessments for cardiovascular response.

	IG			OG			CG			Intermediate				Final				
	Median (interquartile range)									*t* ^a^	*df* ^a^	*p* ^a^	Pairwise comparisons (groups)^b,c^	*t* ^a^	*df* ^a^	*p* ^a^	Pairwise comparisons (groups)^b,c^	Pairwise comparisons (moments)^b,c^
	Initial	Intermediate	Final	Initial	Intermediate	Final	Initial	Intermediate	Final									
SBP (mm Hg)	113 (25)	105 (28)	105 (44)	121.5 (25)	124 (23)	120 (23)	115 (67)	119.5 (42)	113.5 (43)	1.523	2	*p* > 0.05	d	3.117	2	*p* > 0.05	d	initial ≠ final (IG and OG)
DBP (mm Hg)	70 (12)	77.5 (14)	77 (18)	77.5 (43)	80 (43)	79 (27)	80.5 (24)	81.5 (28)	83 (15)	2.338	2	*p* > 0.05	d	3.904	2	*p* > 0.05	d	d
HRrest (mm Hg)	67.5 (15)	68.5 (13)	64 (14)	67.5 (11)	68.5 (10)	68 (17)	85 (18)	86 (14)	88.5 (14)	4.611	2	*p* > 0.05	d	8.516	2	*p* = 0.014	1≠3 (*t* = -2.912; *p* = 0.011	initial ≠ final (CG)
Mean RR (ms)	865.06 (208)	538.83 (302.84	745.89 (317.46)	879.15 (240.81)	880.50 (193.02)	782.94 (349.02)	659.47 (89.74)	566.68 (97.45)	621.09 (614.38)	6.865	2	*p* > 0.05	d	5.432	2	*p* > 0.05	d	pré ≠ intermediate (IG)
SDNN (ms)	35.45 (31.12)	36.86 (61.21)	36.12 (24.48)	32.64 (53.4)	37.08 (55.95)	30.40 (36.63)	24.86 (33.35)	37.43 (32.41)	26.51 (25.96)	1.091	2	*p* > 0.05	d	1.455	2	*p* > 0.05	d	d
Mean HR (ms)	69.47 (16.10)	111.36 (44.45)	80.47 (26.39)	68.25 (18.89)	68.15 (14.62)	77.63 (32.08)	91.01 (11.54)	105.97 (18.16)	95.7 (19.18)	6.865	2	*p* > 0.05	d	3.948	2	*p* > 0.05	d	pré ≠ intermediate (IG)
RMSSD (ms)	31.57 (16.6)	17.94 (14.13)	19.99 (23.11)	31.32 (24.44)	32.79 (37.04)	19.2 (23.39)	12.05 (18.18)	10.04 (17.44)	11.25 (14.52)	3.792	2	*p* > 0.05	d	2.071	2	*p* > 0.05	d	d
pNNxx (%)	8.92 (15.66)	.78 (2.31)	1.91 (15.25)	8.59 (20.47)	9.63 (20.3)	1.85 (10.02)	0 (3.75)	0 (4.78)	0 (4.64)	2.994	2	*p* > 0.05	d	2.712	2	*p* > 0.05	d	d
LF log	5.89 (2.11)	5.5 (3.47)	5.36 (2.3)	5.56 (2.30)	6.24 (3.67)	6 (2.45)	5.37 (2.44)	4.94 (2.74)	4.62 (2.71)	2.226	2	*p* > 0.05	d	1.922	2	*p* > 0.05	d	d
HF log	5.39 (.49)	3.51 (3.45)	4.88 (2.69)	5.84 (2.59)	5.77 (3.12)	4.01 (2.08)	4.26 (1.56)	2.71 (4.29)	4.1 (2.06)	3.703	2	*p* > 0.05	d	1.477	2	*p* > 0.05	d	initial ≠ intermediate (IG)
LF n.u	63.38 (44.14)	89.54 (14.70)	66.07 (13.28)	53.57 (46.25)	74.47 (38.45)	69.28 (26.26)	62.95 (53)	78.71 (17.41)	72.04 (31.79)	3.614	2	*p* > 0.05	d	3.058	2	*p* > 0.05	d	d
HF n.u	36.58 (44.14)	10.44 (14.53)	33.91 (13.36)	36.58 (44.14)	25.51 (38.44)	30.53 (26.26)	37.03 (52.93)	21.21 (17.4)	27.57 (26.74)	3.614	2	*p* > 0.05	d	3.703	2	*p* > 0.05	d	d
LF/HF ratio	1.73 (2.62)	8.57 (8.03)	1.95 (1.12)	1.15 (6.25)	2.91 (2.92)	2.26 (11.71)	1.69 (5.39)	3.71 (4.41)	2.57 (2.16)	3.614	2	*p* > 0.05	d	3.058	2	*p* > 0.05	d	d

IG, indoor group; OG, outdoor group; CG, control group; *t*, test statistic; *df*, degrees of freedom; a, *Kruskal–Wallis*; b, *Friedmann*; c, *Bonferroni correction*; d, No differences detected; SBP, systolic blood pressure; DBP, diastolic blood pressure; HR_rest_, heart rate rest; mean RR, mean of the RR intervals; SDNN, standard deviation of RR intervals; RMSSD, root mean square of consecutive RR intervals; pNN50, percentage of successive RR intervals that differ by more than 50 m; LF, absolute power of the low-frequency band, 0.04–0.15 Hz, in ms2, HF, absolute power of the high-frequency band, 0.15–0.4 Hz, in ms2; LF-to-HF power (LF/HF).

There was a significant difference between assessments in the SBP variable in the IG (initial ≠ final moment; *Bonferroni* corrected: *t* = 2.454; *p* = 0.042; *W* = 0.08) and in the OG (initial ≠ final; *Bonferroni* corrected: *t* = 2.405; *p* = 0.048; *W* = 0.08). Considering DBP, there was a difference between initial and final assessments in the CG (*Bonferroni* corrected: *t* = 2.405; *p* = 0.048; *W* = 0.08). Regarding the mean RR variable, there was a significant difference between assessments in the IG (initial ≠ intermediate; *Bonferroni* corrected: *t* = 2.598; *p* = 0.028; *W* = 0.09). There are differences between assessments in the IG for the mean RR variable (initial ≠ intermediate; *Bonferroni* corrected: *t* = −2.598; *p* = 0.028; *W* = 0.09). Similarly, there was a significant difference between assessments in the IG regarding the HF log (initial ≠ intermediate; *Bonferroni* corrected: *t* = 2.405; *p* = 0.048; *W* = 0.08).

In Table 9, it can be observed the differences between groups and assessments for metabolic status. Considering metabolic status, there were no differences between groups and assessments.

**TABLE 9:** Difference between groups and assessments for metabolic status.

	IG		OG		CG		Final				
	Median (interquartile range)						*t* ^a^	*df* ^a^	*p* ^a^	Pairwise comparisons (groups)^b,c^	Pairwise comparisons (moments)^b,c^
	Initial	Final	Initial	Final	Initial	Final					
Glycemia (mg/dL)	89 (32)	85 (8.5)	98 (29)	94 (23)	87 (53)	83 (53)	*1.172*	*2*	*p* > 0.05	d	d
Cholesterol (mg/dL)	171 (65)	177.5 (66)	199 (66)	192 (64)	170 (78)	202 (118)	*1.795*	*2*	*p* > 0.05	d	d
Triglycerides (mg/dL)	132 (49)	141 (87)	113 (95)	100 (86)	180 (167)	145 (173)	*.453*	*2*	*p* > 0.05	d	d

IG, indoor group; OG, outdoor group; CG, control group; *t*, test statistic; *df*, degrees of freedom; a, *Kruskal–Wallis*; b, *Wilcoxon*; c, *Bonferroni correction*; d, no differences detected.

In Table 10, it can be observed the differences between groups and assessments for neuromuscular capacity. Considering neuromuscular capacity, there were no significant differences between groups and assessments for concentric knee extension at 60°/s for the right and left sides, concentric knee flexion at 60°/s for the right side, concentric knee extension at 120°/s for the right and left sides, and concentric knee flexion at 120°/s for the right side. Considering the intermediate assessment, there was a difference between the IG and CG for concentric knee flexion at 60°/s for the left side (after *Bonferroni correction*: *t* = 2.585; *p* = 0.029; *W* = 0.09). Regarding the final assessment, there was a difference between the IG and CG (after *B. correction*: *t* = 3.145; *p* = 0.005; *W* = 0.11). Differences were also found between groups in the concentric knee flexion at 120°/s left side test when considering the final assessment (IG ≠ CG; *B. correction*: *t* = 2.627; *p* = 0.026; *η* ^*2*^ = 0.329).

**TABLE 10:** Difference between groups and assessments for neuromuscular capacity.

	IG			OG			CG			Intermediate				Final				
	Median (interquartile range)									*t* ^a^	*df* ^a^	*p* ^a^	Pairwise comparisons (groups)^b,c^	*t* ^a^	*df* ^a^	*p* ^a^	Pairwise comparisons (groups)^b,c^	Pairwise comparisons (moments)^b,c^
	Initial	Intermediate	Final	Initial	Intermediate	Final	Initial	Intermediate	Final									
PT cc KE 60°/s right (N.M)	59.8 (42.5)	79 (63.9)	74.85 (23.8)	55.2 (73.4)	67.3 (76)	75.8 (61.8)	51.1 (43.7)	62.7 (30.6)	55.5 (71.1)	1.3	2	*p* > 0.05	d	1.67	2	*p* > 0.05	d	d
PT cc KE 60°/s left (N.M)	73.2 (49.8)	78 (56.9)	80.35 (30.1)	44.7 (72.5)	50.2 (82)	53.9 (54.8)	58.6 (38.2)	57.9 (59)	49.6 (60.2)	2.131	2	*p* > 0.05	d	4.736	2	*p* > 0.05	d	d
PT cc KF 60°/s right (N.M)	35.3 (27.9)	35.3 (39.9)	34.2 (21.6)	20.5 (44.5)	49.6 (47.3)	25.5 (41.1)	16.35 (14)	23 (12.1)	18.9 (25)	2.703	2	*p* > 0.05	d	5.818	2	*p* > 0.05	d	d
PT cc KF 60°/s left (N.M)	38.2 (17.6)	41.85 (13.33)	51.15 (13.77)	16 (34.65)	34.7 (37.38)	23.4 (33.42)	16.4 (24.05)	27.2 (26.88)	18.3 (11.98)	6.772	2	*p* = 0.034	1≠3 (*t* = 2.585); *p* = 0.029	9.943	2	*p* = 0.007	1≠3 (*t* = 3.145); *p* = 0.005	d
PT cc KE 120°/s right (N.M)	48.9 (49.78)	51 (−.06)	57.15 (29.15)	24.95 (49.25)	40.9 (47.45)	33.45 (43.45)	31.65 (18.66)	29.75 (32.6)	35.5 (25.73)	2.072	2	*p* > 0.05	d	4.282	2	*p* > 0.05	d	d
PT cc KE 120°/s left (N.M)	35.7 (42.9)	42.9 (27.4)	59.6 (45.2)	14 (53.2)	29.7 (36.25)	24.8 (25)	36.7 (26.05)	41.3 (39.55)	47.7 (44.45)	2.503	2	*p* > 0.05	d	4.059	2	*p* > 0.05	d	d
PT cc KF 120°/s right (N.M)	23.1 (19.65)	23.1 (11.85)	28.1 (7.75)	16 (36.9)	35.2 (34.15)	8.5 (33.2)	11.2 (12.45)	15.6 (9.8)	17.9 (10.9)	5.28	2	*p* > 0.05	d	2.9	2	*p* > 0.05	d	d
PT cc KF 120°/s left (N.M)	23.3 (33.6)	29.6 (14.15)	36.5 6)	6.8 (31.15)	24.7 (25.6)	13.4 (28.85)	11.3 (8.35)	15.6 (34.1)	17.3 (11.05)	3.631	2	*p* > 0.05	d	7.577	2	*p* = 0.023	1≠3 (*t* = 2.627); *p* = 0.026	d
3 kg medicine ball throw (m)	2.12 (.93)	2.4 (.7)	2.45 (.73)	1.7 (1.47)	1.95 (1.15)	2.45 (1.58)	2.38 (1.01)	2.3 (.91)	2.07 (1.23)	1.52	2	*p* > 0.05	d	4.766	2	*p* > 0.05	d	initial ≠ final (IG)—*t* = -2.887; *p* = 0.012; initial ≠ final (OG)—*t* = -3.074; *p* = 0.006; initial ≠ final (CG)—*t* = 2.539; *p* = 0.033
manual dynamometer (kg)	23.05 (7.67)	31.35 (10.48)	24.85 (9.9)	16.3 (11.77)	16.5 (16.38)	21.8 (17.83)	13.9 (8.7)	14.5 (11)	13.1 (10.4)	5.106	2	*p* > 0.05	d	6.593	2	*p* = 0.043	1≠3 (*t* = 2.413); *p* = 0.047	initial ≠ final (IG)—*t* = -3.175; *p* = 0.004; initial ≠ final (OG)—*t* = -2.94; *p* = 0.01; intermediate ≠ final (OG)—*t* = -2.673; *p* = 0.023

IG, indoor group; OG, outdoor group; CG, control group; *t*, test statistic; *df*, degrees of freedom; a, *Kruskal–Wallis*; b, *Friedmann*; c, *Bonferroni correction*; d, No differences detected.

At the initial assessment, no differences were found between groups in the 3 kg medicine ball throw test (*p* ≥ 0.05). Similarly, there was no difference between the intermediate and final assessments. Considering assessments, there were significant differences in the IG (initial ≠ final; *Bonferroni corrected*: *t* = −2.887; *p* = 0.012; *W = 0.1*), OG (initial ≠ final; *B. corrected*: *t* = −2.887; *p* = 0.012; *W = 0.10*), and CG (initial ≠ final; *B. corrected*: *t* = 2.59; *p* = 0.033; *W = 0.09*), respectively.

Regarding the hand grip force test, there were no differences between groups at the initial and intermediate assessments. Differences were found between groups at the final assessment (*p* = 0.043). After *B. correction*, these differences were only observed between the IG and CG (*t* = 2.413; *p* = 0.047; *W* = 0.08). There were also statistical differences between assessments in the IG (initial ≠ final; *B. corrected*: *t* = −3.175; *p* = 0.004; *W* = 0.02) and the OG (initial ≠ final; *B. corrected*: *t* = −2.94; *p* = 0.01; *W* = 0.10 and intermediate ≠ final; *B. corrected*: *t* = −2.673; *p* = 0.023; *W* = 0.09).

## 4 Discussion

The present non-randomized experimental study aimed to assess the effects of 24-week exercise programs on health indicators and neuromuscular capacity in individuals with IDD. To our knowledge, this is the first study to evaluate the effects of an exercise program on the evaluated variables, considering the different contexts. A low-cost outdoor intervention appears to be effective in reducing fat mass and an indoor intervention seems to be a good method to develop neuromuscular capacity. Thus, we partially accept the hypotheses i, ii, v, vi, vii, and viii.

### 4.1 Anthropometry and body composition

No significant differences were found between groups for all variables. However, the fat mass decreased in the OG for both the intermediate and final assessments when compared to the initial assessment. Although the differences were not significant, this group (OG) also showed a decrease in WC after 12 weeks of training, reducing BMI and increasing muscle mass (12 and 24 weeks). The outdoor intervention seems to be more effective for promoting body composition changes. Intervention studies in the IDD population, besides being limited, have different approaches and lack meta-analysis studies to identify which exercise prescription has the greatest effects on the variables assessed A possible justification for the lack of significant differences in the remaining variables of body composition may be due to the fact that variables such as diet/calorie intake were not controlled in the present study, although being known that the average value of calories intake is higher in the IDD population (Hoey et al., 2017). Also, individuals with IDD (namely, Down Syndrome) have been shown to have total energy expenditure values lower than those without disability (Polfuss et al., 2018; Ohwada et al., 2021), which negatively affects the ratio of caloric intake to energy expended (Polfuss et al., 2018). Future approaches should take the balance of calorie intake (nutrition) and energy expenditure (physical activity and basal metabolism) into consideration, in the sense that intake should not exceed expenditure. In addition, family members/guardians/caregivers often overestimate the amount of energy expended through physical activity and the amount they should ingest, contributing to overrating the individual’s energy needs (Segal et al., 2016). At the same time, they will also be able to provide food support according to the needs/quantities identified. A multidimensional (exercise + diet), literate approach for family/guardians/caretakers/individuals with IDD themselves should be considered in future studies.

Furthermore, a 24-week exercise program with a twice-a-week frequency may not be enough to produce changes in body composition. In this sense, future studies should implement a longer duration program or have a higher weekly frequency as suggested by Yu et al. (2022). Their study found significant differences in BMI (initial vs. final moment: 28.16 ± 3.69 vs. 27.5 ± 3.97; Control group: 27.37 ± 3.99 vs. 28.05 ± 3.75) using a 36 weeks program (2 sessions per week). However, despite exercising two or three times per week, IDD individuals may still exhibit high rates of sedentary behavior, compromising the positive impact of a physical exercise program.

The fact that the amount of daily life physical activity practiced outside the intervention programs was not measured and, specifically for the control group, may also have influenced the results and may explain the lack of significant differences. For more robust results, it is suggested that future studies quantify the daily physical activity of all participants in the study throughout the intervention program (e.g., using accelerometers, pedometers; and IPAQ) (Ptomey et al., 2017).

Future studies should also analyze gender differences.

### 4.2 Metabolic status

Taking into account metabolic status there were no significant differences between groups and assessments, similar to the results found by Boer and Moss (2016), where the authors also prescribed and implemented two different interventions for two groups: continuous aerobic training or interval training three times a week for 12 weeks. The lack of significant differences in glycemia and triglycerides can be explained by the fact that these values were already within the normative standards. As for the cholesterol values, which were high in the OG, it was possible to observe a reduction with exercise, although it was not significant and far from the reference values. Aerobic interval training is a good method for promoting a change in these variables in the IDD population (Boer et al., 2014), so future studies implementing the two training programs used for this study (indoor and outdoor) should increase the duration of the first bout of exercises, or only perform the shuttle run. Calders et al. (2011) when prescribing a combined exercise program, found a significant difference in cholesterol variables. However, they prescribed more aerobic exercise volume than in the present study, which may be crucial for the positive effects.

### 4.3 Cardiovascular response

After 24 weeks the IG and OG decreased SBP, showing that both the intervention programs performed were beneficial for improving the risk factors of cardiovascular disease. Similarly, (Boer and Moss, 2016), when prescribing an interval exercise program found a significant difference in SBP at the final intervention (initial vs. final: 124 ± 10 mm Hg vs. 113 ± 8 mm Hg; CG: 118 ± 10 mm Hg vs. 119 ± 10 mm Hg). The practice of any method of physical exercise seems to enhance the reduction of SBP.

Compared to the CG, the intervention in the gym environment seems to have been more effective in reducing HRrest. Moreover, a previous meta-analysis highlighted that resistance training is an effective method for reducing HRrest (Reimers et al., 2018). The fact that, for the IG, the control of the intensity for the aerobic exercise followed a more objective method can explain the higher impact of the exercise program on the HRrest of the IG compared to the OG. A significant increase in HRrest after 24 weeks was also observed in the CG. The effect only occurs over a few months on a mean of 3 months with three sessions per week. In addition, the age of the participants may be negatively associated with a decrease in exercise-induced HRrest (Reimers et al., 2018). Furthermore, a high HRrest rate increases mortality for all causes by 17% (Aune et al., 2017). The mechanisms of this relationship are still not completely known. Possible mechanisms may be endothelial dysfunction, reduced artery compliance and distensibility, and, consequently, increased arterial wall stress and elevated pulse wave velocity, which is associated with increased afterload and systemic hypertension (Tadic et al., 2018). It is important that assessments and interventions should always take place at the same time since the time of the day has a considerable influence on heart rate patterns (Waninge et al., 2013).

Significant differences were found in increasing mean HR and decreasing mean RR and HFlog in the IG after 12 weeks. A higher state of excitation on the data collection dates or some performed activity of higher intensity that was not controlled/reported before data collection may have influenced the results. The literature shows that a change in mean RR may demonstrate that training induces bradycardia at rest accompanied by an increase in cardiac vagal modulation. Through this mechanism, physical training may be able to exert an antiarrhythmic effect (Sandercock et al., 2005), however, such results were not found in the present study. Furthermore, individuals with IDD may present autonomic dysfunction and HRV values lower than in populations without disability (Chang et al., 2012), which may be influenced by factors such as obesity, low physical fitness, and age (Mendonca et al., 2013). We also highlight the fact that all participants in this study had SDNN values lower than 50 m, which is related to a higher risk of cardiovascular diseases (Kleiger et al., 2005).

### 4.4 Neuromuscular capacity

Significant differences were found between groups, at the intermediate assessment, namely, for knee concentric flexion peak force at 60 °/s and for the left side test. Differences were also found at both intermediate and final assessments, between the IG and CG. Additionally, significant differences were also found between groups for knee concentric flexion peak force at 120°/s left test, at the final assessment. The results are in agreement with the study by Ko et al. (2012), where the authors also found significant differences in the left lower limb. Such results may be related to hypothetical differences in strength between dominant and non-dominant limbs. Furthermore, the authors also found a significant difference in the extension test for both limbs, something that did not occur in our study as only a slight difference was observed. Additionally, Cowley et al. (2011) found improvements in the bilateral flexor and extensor tests when prescribing an exercise program of only resistance training. Compared to our prescription, the author prescribed the load as a function of 10 maximum repetitions for each exercise. Although a slight increase was seen, the OG showed no significant differences in strength gains when comparing by groups or assessment times. Compared to the CG, the OG seemed to be more effective in increasing the isokinetic force of the hamstring muscles.

At the same time, there were no differences at the intermediate and final assessments. Considering the 3 kg medicine ball throw test, there was a difference only between the initial and final assessments in all groups. For the manual dynamometer test, there were differences between the IG and the CG at the final assessment. In the same way, the OG significantly improved the results in all assessments, and the IG improved at the last assessment. The combined exercise program seems to have been fundamental for these results as a previous study found no benefit in terms of strength of the upper limbs when using a continuous or an interval aerobic exercise intervention program (Boer and Moss, 2016). These results are important as muscle strength is an important variable in the functionality, success in performing activities of daily living, and independence of individuals with IDD (Cowley et al., 2010).

### 4.5 Adverse effects

A few adverse events were registered including muscle pain and fatigue as a result of the defined intensity value. On rest days, those participants were sent to the physiotherapy office.

### 4.6 Strengths and limitations

Our study presents two exercise programs that appear to be effective and can mitigate/attenuate the barriers that this population has to exercise and being active. These two programs can be implemented by any institution/organization, taking into consideration the economic and environmental possibilities, and seem to be feasible for any individual with IDD. The OG exercise program can also be performed with body weight only, using bottles with sand or other low-cost materials (Ferreira et al., 2022).

Although our study generally showed promising results, several limitations must be acknowledged when analyzing the results. The lack of monitoring of the amount of physical activity performed by all participants in daily life activities is the main limitation of this study, in the sense that it may have negatively impacted our results. We could not ascertain the impact of exercise on IDD levels because the recruitment institution did not have information regarding all the participants’ IQs. The fact that the exercise sessions were conducted by two accredited instructors may also have limited our results, since the quality/quantity of support provided in explanation, demonstration, support, and feedback could not have been the same for all individuals, leading to the fact that the rest time between sets may have been longer than prescribed. Due to logistical constraints and the daily operation of the recruitment institutions, it was not possible to conduct a randomized controlled trial. Finally, the different prescription/control of strength training between the programs does not allow for a clear comparison.

## 5 Conclusion

A low-cost outdoor intervention in contact with nature appears to be effective in reducing fat mass. None of the interventions showed significant differences in the metabolic status variables. Similarly, the difference in cardiovascular response was not clear and robust. Finally, an indoor intervention using weight-training machines seems to be a good method to promote neuromuscular capacity.

Our study presents two comprehensive exercise programs that appear to be effective and may mitigate/attenuate the barriers that individuals with IDD face when attempting to engage in sports, namely, the lack of tailored exercise programs and the high financial cost of practice.

## Data Availability

The raw data supporting the conclusions of this article will be made available by the authors, without undue reservation.
